# A comparative study of clustering methods on gene expression data for lung cancer prognosis

**DOI:** 10.1186/s13104-023-06604-8

**Published:** 2023-11-08

**Authors:** Jason Z. Zhang, Chi Wang

**Affiliations:** 1 Wake Forest University, Winston-Salem, NC United States of America; 2 Markey Cancer Center, University of Kentucky, Lexington, KY USA

**Keywords:** Clustering, Gene expression, Prognosis, Comparison

## Abstract

Lung cancer subtyping based on gene expression data is important for identifying patient subgroups with differing survival prognosis to facilitate customized treatment strategies for each subtype of patients. Unsupervised clustering methods are the traditional approach for clustering patients into subtypes. However, since those methods cluster patients based only on gene expression data, the resulting clusters may not always be relevant to the survival outcome of interest. In recent years, semi-supervised and supervised methods have been proposed, which leverage the survival outcome data to identify clusters more relevant to survival prognosis. This paper aims to compare the performance of different clustering methods for identifying clinically prognostic lung cancer subtypes based on two lung adenocarcinoma datasets. For each method, we clustered patients into two clusters and assessed the difference in patient survival time between clusters. Unsupervised methods were found to have large logrank p-values and no significant results in most cases. Semi-supervised and supervised methods had improved performance over unsupervised methods and very significant p-values. These results indicate that unsupervised methods are not capable of identifying clusters with significant differences in survival prognosis in most cases, while supervised and semi-supervised methods can better cluster patients into clinically useful subtypes.

**Supplementary Information:**

The online version contains supplementary material available at 10.1186/s13104-023-06604-8.

## Introduction

The leading cause of cancer death for both men and women is lung cancer [1]. However, risk levels vary between patients, so it is important to identify clinically prognostic subtypes of lung cancer with different risk levels and target higher risk subtypes for more aggressive treatment [2, 3]. Gene expression data may be used for this screening, as expression levels of certain genes are associated with cancer prognosis [4]. In addition to lung cancer, molecular subtypes have been widely investigated in many other cancer types, such as breast cancer [5] and colorectal cancer [6]. We refer the readers to [7–10] for comprehensive reviews of cancer subtyping.

Traditionally, there have been various unsupervised clustering algorithms used for this purpose. Unsupervised clustering methods treat all features equally, without considering their significance to the clinical outcome. As data typically contain multiple underlying structures, e.g. a set of documents may be clustered based on authorship, topic, or writing style [11], the results of unsupervised clustering can be driven by one dominant structure, or a mixture of several structures. There is no guarantee that the structures identified by unsupervised clustering is relevant to the outcome of interest [12]. In cancer subtyping studies, it has been shown that the subtypes identified by unsupervised clustering may sometimes be related to cell-of-origin [13] or histology [14] rather than patient’s survival time. Therefore, although subtypes from unsupervised clustering may be useful for investigating the biology of cancer, they are not always associated with clinical prognosis [12]. To combat this, semi-supervised methods, which attempt to consider both expression and clinical data, have been proposed [15]. These methods select genes that are more closely related to clinical outcome for clustering input [15]. Recently, supervised clustering methods have also been proposed, such as survClust [12], ogClust [16], and [17]. These methods directly incorporate clinical outcome information into the clustering process to ensure that the resulting clusters are clinically relevant.

In this paper, we compare the performance of these three types of clustering algorithms for identifying prognostic subtypes in lung cancer, providing a guidance on future applications of these approaches. The two datasets used are from patients with lung adenocarcinoma, one of the most common types of lung cancer [18]. We will apply each clustering method to identify subtypes and evaluate the performance based on the prognostic difference between subtypes.

## Methods

### Data

Two datasets were considered. The first dataset used was the lung adenocarcinoma dataset from The Cancer Genome Atlas Research Network (TCGA-LUAD) [18], which included patient gene expression data (transcript per million (TPM) values, 56,716 features), as well as clinical data, such as smoking history, cancer stage, patient survival time, patient censoring event (patient status at the time of leaving the study), patient age, and patient sex. The second dataset was from a study conducted by Shedden et al. [19], which included similar data variables, with fewer gene expression features (normalized microarray data with 22,283 features). Patients without both clinical and gene expression data were excluded. Furthermore, duplicate entries for patients were excluded, along with patients with survival time of 0. After applying a 2-component principal component analysis (PCA), outliers were found and removed. All gene expression data were scaled from 0 to 1 using min-max scaling. In the TCGA-LUAD dataset, 483 patients remained after data preparation, while in the Shedden dataset, 346 patients remained. A summary of patient characteristics is provided in Supplementary Table 1.

### Clustering methods

The clustering methods tested were unsupervised methods, semi-supervised methods, and outcome-guided methods. For unsupervised methods, k-means (KM) [20], Gaussian mixture (GM), and agglomerative clustering (AC) were considered. Implementations of KM, GM, and AC models were provided by Scikit-learn [21]. In addition, we also included a Consensus clustering method based on a voting consensus of KM, GM, and AC clustering results, which follows the idea of [22]. Specifically, the method with the best agreement with the other two was selected as the reference, with the other two methods’ results adjusted to this reference and a best-of-3 voting deciding the final cluster of each patient. Note that our approach to select the reference method differed from that in [22] because we did not leverage any clinical information in order to make the method unsupervised. Strategies to include the clinical outcome variable in combination with Consensus clustering or other unsupervised methods to enable semi-supervised learning are described in the next paragraph. Prior to clustering analysis, a PCA was performed to reduce the dimension of features. The PCA considered gene expression data, as well as data on sex and age. The top principal components (PCs) sufficient to explain 95% of the variance in the data along with two important clinical variables, i.e. cancer stage and smoking status, were used as features in the clustering analysis.

For semi-supervised methods, feature selection methods were first used to select features, and these selected features were then used by clustering methods to cluster patients. Specifically, feature selection methods included Cox proportional-hazards regression [23] and random survival forests (RF) [24]. Implementation of Cox regression was provided in Python by lifelines [25], and the implementation of RF was provided in R [26]. Each feature selection model used patient survival time as the outcome variable and considered the same set of features as unsupervised methods. Based on each feature selection method, 20 features were selected for consideration in clustering. For the Cox regression, each feature was fitted univariately to the survival outcome, and the features with the lowest p-values were selected. For RF, the top 20 variables selected in a minimal-depth search were used. Based on the feature-selected data, unsupervised clustering methods, including KM, GM, AC, and Consensus, were applied. We considered the following eight combinations of feature selection and clustering methods for each dataset (TCGA or Shedden): Cox-KM, Cox-GM, Cox-AC, Cox-Consensus, RF-KM, RF-GM, RF-AC, and RF-Consensus.

For supervised methods, survClust (SC) [12] was considered. Patient survival time was used as the target value, and the same set of features as unsupervised methods were considered. survClust was trained using cross-validation, with final clustering results based on the consensus after 10 rounds of 3-fold cross-validation, as suggested by the authors [12].

All methods were set to cluster into 2 clusters. The number of clusters was selected to evaluate the ability of the methods to separate patients into good and bad prognostic subtypes. Method parameters that were changed from defaults are provided in Supplementary Table 2. Each method was run 200 times and averaged results were reported to reduce variation in performance due to random seeds, etc. Note that survClust had 200 trials of the 10-round 3-fold cross-validation run.

### Evaluation

To evaluate the performance of the methods, Kaplan-Meier survival curves [27] were plotted to visualize the difference in survival time distribution between the two clusters predicted by a clustering method.

Utilizing the survival curves, p-values based on logrank tests were calculated to characterize the performance of the methods. A lower p-value indicates a better-performing method. A significance threshold of p = 0.05 was set to indicate the best performing methods. Since all methods were run 200 times, the average p-value is used to represent the performance of a method. Furthermore, Adjusted Rand Index (ARI) [28] was used to assess the similarity between the clusters of two methods. Specifically, each of the 200 replicates from one method was compared to each of the 200 replicates from the other method to obtain an ARI value. The averaged ARI of those 40,000 values was reported.

## Results

**Fig. 1:**
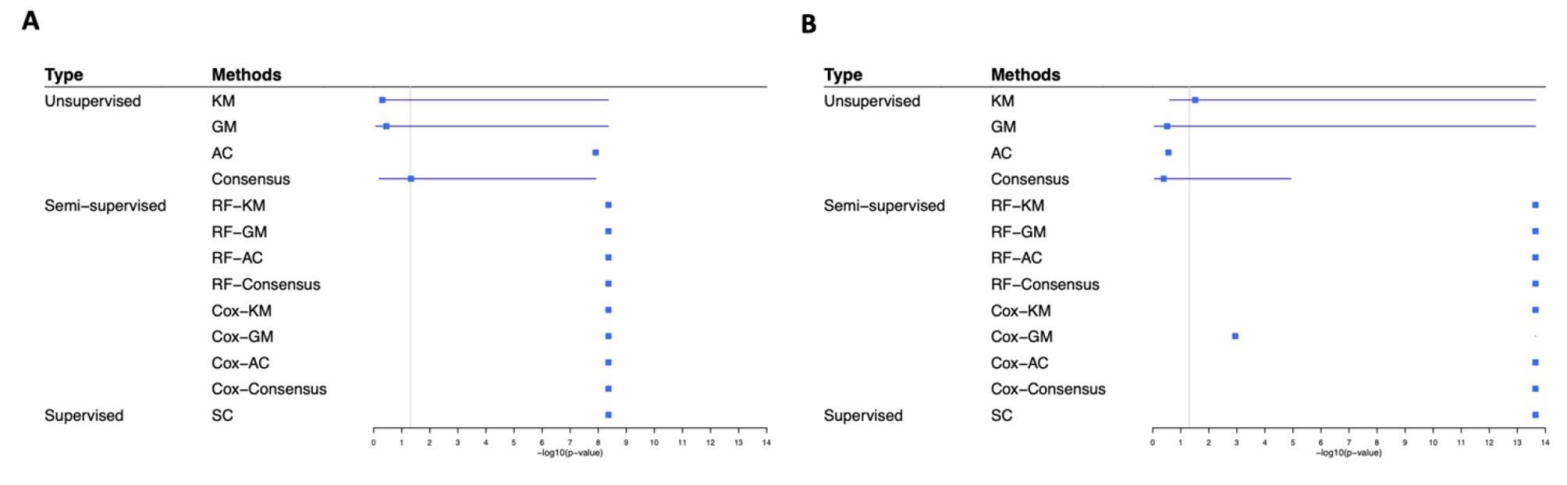
Comparison of p-values from different clustering methods. Note: These forest plots display -log_10_ p-value for comparing survival distribution between the two clusters identified by each clustering method based on **(A)** TCGA-LUAD or **(B) **Shedden data. The methods are grouped based on clustering algorithms, with unsupervised methods on top, semi-supervised methods in the center, and survClust (SC), the supervised method, on the bottom. Semi-supervised methods and SC present the best performance of all the methods. The solid square indicates -log_10_(mean p-value) and horizonal line indicates the corresponding 95% CI over 200 trials for each method. The significance threshold of p = 0.05 is marked with a vertical line, with significant values to the right of the line

We applied unsupervised, semi-supervised, and supervised clustering methods to the TCGA-LUAD [18] and Shedden [19] datasets to investigate which methods could identify clinically prognostic subtypes. Based on each method, we identified two clusters, and compared the survival time distributions of the two clusters of patients. Table 1; Fig. 1 summarize the overall results for every method, with mean, standard deviation, and 95% CI based on 200 trials. Supplementary Figs. 1 and 2 display the Kaplan-Meier survival curves of the two clusters based on a representative trial of each method for TCGA-LUAD and Shedden data, respectively.

**Table 1:** Overall results of different clustering methods

		TCGA-LUAD			Shedden		
Type	Method	Mean p-value	Standard Deviation	95% CI	Mean p-value	Standard Deviation	95% CI
Unsupervised	KM	0.493	0.107	(4.32E-09, 0.516)	3.06E-02	8.46E-02	(2.25E-14, 0.244)
	GM	0.355	0.293	(4.32E-09, 0.857)	0.308	0.260	(2.25E-14, 0.892)
	AC	1.23E-08	0	(1.23E-08, 1.23E-08)	0.276	5.55E-17	(0.276, 0.276)
	Consensus	4.69E-02	0.167	(1.23E-08, 0.648)	0.408	0.404	(1.19E-05, 0.876)
Semi-Supervised	RF-KM	4.32E-09	1.42E-23	(4.32E-09, 4.32E-09)	2.25E-14	6.83E-28	(2.25E-14, 2.25E-14)
	RF-GM	4.32E-09	1.55E-23	(4.32E-09, 4.32E-09)	2.25E-14	6.83E-28	(2.25E-14, 2.25E-14)
	RF-AC	4.32E-09	0	(4.32E-09, 4.32E-09)	2.25E-14	0	(2.25E-14, 2.25E-14)
	RF-Consensus	4.32E-09	1.85E-11	(4.32E-09, 4.32E-09)	2.25E-14	6.88E-28	(2.25E-14, 2.25E-14)
	Cox-KM	4.32E-09	1.48E-23	(4.32E-09, 4.32E-09)	2.25E-14	6.79E-28	(2.25E-14, 2.25E-14)
	Cox-GM	4.32E-09	1.56E-23	(4.32E-09, 4.32E-09)	1.14E-03	1.15E-02	(2.25E-14, 2.25E-14)
	Cox-AC	4.32E-09	0	(4.32E-09, 4.32E-09)	2.25E-14	0	(2.25E-14, 2.25E-14)
	Cox-Consensus	4.32E-09	1.53E-23	(4.32E-09, 4.32E-09)	2.25E-14	6.91E-28	(2.25E-14, 2.25E-14)
Supervised	SC	4.32E-09	2.71E-22	(4.32E-09, 4.32E-09)	2.25E-14	1.24E-27	(2.25E-14, 2.25E-14)

Note: This table displays the overall clustering results, providing p-values, standard deviations for p-values, and 95% confidence intervals (CIs). Methods are organized by type (unsupervised, semi-supervised, and supervised). Results are summarized from 200 trials for each method. Agglomerative clustering results, except for RF-AC, do not have a standard deviation or confidence interval, as they are not affected by random initial values and thus are the same across 200 trials. The 95% CI was calculated based on the 2.5 and 97.5 percentile of p-values from 200 trials

Unsupervised methods presented non-significant differences (p-value > 0.05) in patient survival time between clusters in most cases, except for the AC and Consensus methods on TCGA-LUAD data and the KM method on Shedden data (Table 1; Fig. 1). The Kaplan-Meier survival curves for those non-significant cases also did not present strong separation between clusters (Supplementary Figs. 1 and 2). Therefore, the unsupervised clustering methods failed to identify clinically meaningful prognostic subtypes in most cases.

As for semi-supervised methods, both random forest- and Cox Regression-selected models yielded highly significant differences in survival time between clusters for both datasets (Table 1; Fig. 1). The corresponding Kaplan-Meier survival curves also showed strong separation between clusters (Supplementary Figs. 1 and 2).

The supervised clustering survClust method also presented a highly significant difference between clusters for both datasets (Table 1; Fig. 1). It had a strong separation between clusters, which are clearly distinguishable in the survival curves (Supplementary Figs. 1 and 2).

**Fig. 2:**
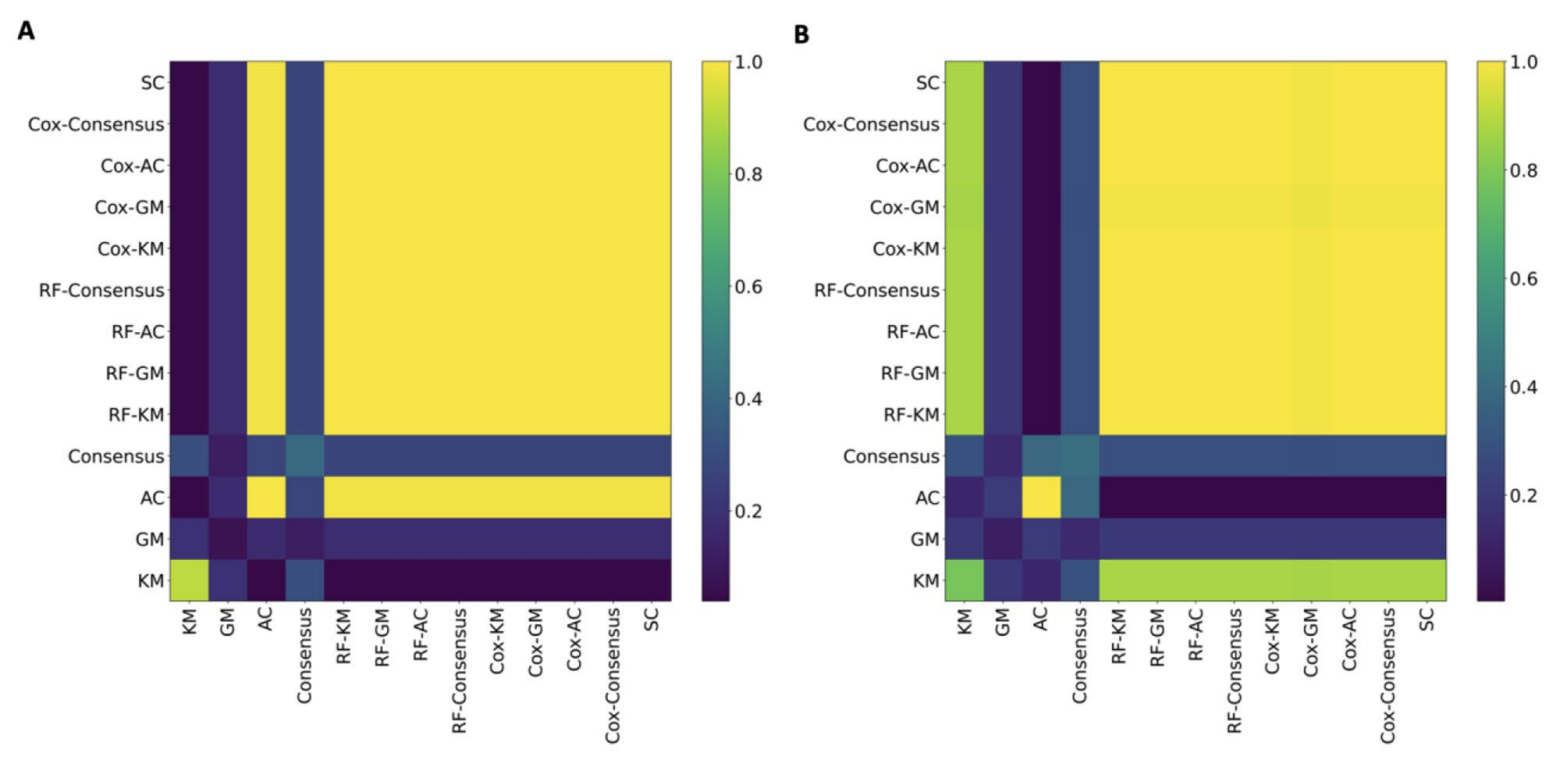
Evaluation of the consistency of clustering results from different clustering methods. Note: The heatmaps present the ARI for each pair of clustering methods based on **(A)** TCGA-LUAD and **(B)** Shedden data. The results present unsupervised methods first, followed by semi-supervised and supervised

To assess the consistency in clustering results across different methods, we calculated the ARI for each pair of methods. As shown in Fig. 2, the clustering results from semi-supervised and supervised methods are highly consistent with each other, and are dissimilar to the results from unsupervised methods (except for AC with TCGA-LUAD data). Therefore, semi-supervised and supervised methods were able to obtain subtypes distinct from those obtained from unsupervised methods.

The analyses above performed clustering using PCs, which is a frequently used approach to reduce the dimension of features but may introduce biases. To make a more unbiased evaluation, we performed a separate set of analyses using all the original features instead of PCs based on TCGA-LUAD data. Results are provided in Supplementary Tables 3 and Supplementary Figs. 3–5. Due to the large number of original features, survClust was unable to generate clustering results. Unsupervised methods and semi-supervised methods with random forest for feature selection also yielded less significant results. Specifically, all p-values from unsupervised methods were greater than 0.05, and only the p-value from RF-GM was smaller than 0.05. Semi-supervised methods with Cox regression for feature selection appeared to be less affected by the switch from PCs to original features, with all p-values remaining highly significant.

## Discussion

Our study showed that unsupervised clustering methods, regardless of the specific clustering algorithm, were unable to identify lung cancer subtypes with different prognosis in many cases. In contrast, semi-supervised and supervised clustering methods were able to identify subtypes with significant difference in patient survival time. Therefore, when the purpose of clustering is to identify prognostic subtypes, supervised and semi-supervised methods would be preferred.

The supervised method, survClust, is unable to converge with large amounts of features, and some measure of feature reduction, such as PCA or a feature selection algorithm, may be required before clustering. It is also more computationally expensive than the other methods tested. Both supervised and semi-supervised methods require the outcome of patients to cluster, so unsupervised methods may be required in a study without outcomes already available, although the clinical interpretation of the clustering results can be ambiguous.

Another item of note is that PCA can improve clustering results. Based on our analysis of TCGA-LUAD data, the analyses based on PCs (Table 1; Fig. 1) yielded smaller p-values than the analyses based on all the original features (Supplementary Tables 3 and Supplementary Fig. 4) for all unsupervised and semi-supervised methods. This is likely due to the reduction in noise that PCA provides.

### Limitations

Our study focuses on gene expression features for the identification of lung cancer prognostic subtypes. It has been well-documented that other molecular features, such as single nucleotide variants, copy number variations, and fusions, are also associated with lung cancer prognosis [10, 28]. Future studies that include those features will provide a more comprehensive assessment and comparison of the performance of different clustering methods. In addition, simulation studies, where there are known “true” clusters, may also need to be conducted to further elucidate the strength and weakness of different clustering methods.

### Electronic supplementary material

Below is the link to the electronic supplementary material.

Supplementary Material 1

## Data Availability

The TCGA-LUAD dataset analyzed during the current study is available in the Genomic Data Commons of the National Cancer Institute, https://gdc.cancer.gov. The Shedden et al. dataset is available in the Gene Expression Omnibus of the National Cancer for Biotechnology Information, https://www.ncbi.nlm.nih.gov/geo/query/acc.cgi?acc=GSE68465.

## Abbreviations

TCGA-LUAD: The Cancer Genome Atlas Research Network lung adenocarcinoma dataset
TPM: Transcript per million
PCA: Principal component analysis
KM: k-means
GM: Gaussian mixture
AC: Agglomerative clustering
PC: Principal component
RF: random survival forests
SC: survClust
ARI: Adjusted Rand Index
CI: Confidence interval
