# Supplementary material for: A comparative study of clustering methods on gene expression data for lung cancer prognosis
Source: BMC Res Notes. 2023 Nov 8;16:319. doi: 10.1186/s13104-023-06604-8 (PMC10630994; doi:10.1186/s13104-023-06604-8)
Supplement: Supplementary file 1 — Supplementary Material 1 [file 13104_2023_6604_MOESM1_ESM.docx]

**Supplementary Materials**

**Supplementary Table 1**

*Demographic and clinical characteristics of TCGA-LUAD and Shedden cohorts*

| **Characteristic** | **TCGA-LUAD**  **(n=483)** | **Shedden**  **(n=346)** |
| --- | --- | --- |
| Age - median (range) | 66 (33, 88) | 65 (35, 87) |
| Sex - no.(%) |  |  |
| Female | 263 (54.5) | 186 (53.8) |
| Male | 220 (45.5) | 160 (46.2) |
| Tumor Stage - no.(%) |  |  |
| I | 333 (68.9) | 298 (86.1) |
| II | 150 (31.1) | 48 (13.9 |
| III and IV |  |  |
| Smoking Status - no.(%) | 263 (54.5) | 217 (62.7) |
| Smoker | 116 (24.0) | 80 (23.1) |
| Non-smoker | 104 (21.5) | 49 (14.2) |

**Supplementary Table 2**

*A summary of parameters for clustering method. Only parameters different from the default values are presented.*

| Algorithm | Parameters changed from default |
| --- | --- |
| k-means (sklearn.cluster.KMeans) | n_clusters = 2 |
| Gaussian Mixture (sklearn.mixture.GaussianMixture) | n_components = 2 |
| Agglomerative Clustering (sklearn.cluster.AgglomerativeClustering) | n_clusters = 2 |
| Random Survival Forest  (var.select) | method = "md"  nrep = 100 |
| Cox Regression  (CoxPHFitter) | penalizer = 0.1 |
| Cox Regression  (CoxPHFitter.fit) | step_size = 0.5 |
| survClust  (cv.survclust) | k = 2 |


**Supplementary Table 3**

*Overall results of different clustering methods based on TCGA-LUAD dataset with all the original features.*

| *Type* | Method | Mean P-value | Standard Deviation | 95% CI |
| --- | --- | --- | --- | --- |
| *Unsupervised* | KM | 0.685 | 0.173 | (0.401, 0.958) |
|  | GM | 0.683 | 0.240 | (3.83E-02, 0.990) |
|  | AC | 0.124 | 2.78E-17 | (0.124, 0.124) |
|  | Consensus | 0.481 | 0.311 | (7.46E-02, 0.988) |
| *Semi-Supervised* | RF-KM | 0.561 | 0.276 | (4.33E-03, 0.873) |
|  | RF-GM | 8.40E-03 | 5.79E-02 | (4.90E-16, 2.53E-02) |
|  | RF-AC | 0.569 | 0.265 | (0.325, 0.873) |
|  | RF-Consensus | 0.279 | 0.321 | (2.99E-06, 0.873) |
|  | Cox-KM | 3.96E-16 | 6.95E-16 | (1.35E-16, 2.50E-15) |
|  | Cox-GM | 1.19E-09 | 3.57E-10 | (1.93E-11, 1.29E-09) |
|  | Cox-AC | 8.58E-14 | 0 | (8.58E-14, 8.58E-14) |
|  | Cox-Consensus | 3.44E-10 | 7.33E-11 | (3.01E-15, 3.60E-10) |

Note: This table displays the overall clustering results, providing p-values, standard deviations for p-values, and 95% confidence intervals. Methods are organized by type (unsupervised, semi-supervised, and supervised). Results are summarized from 200 trials for each method. Agglomerative clustering results, except for RF-AC, do not have a standard deviation or confidence interval, as they are not affected by random initial values and thus are the same across 200 trials. The 95% CI was calculated based on the 2.5 and 97.5 percentile of p-values from 200 trials.

**Supplementary Figure 1**

*Survival curves for different clustering methods based on TCGA-LUAD data with PC features.*


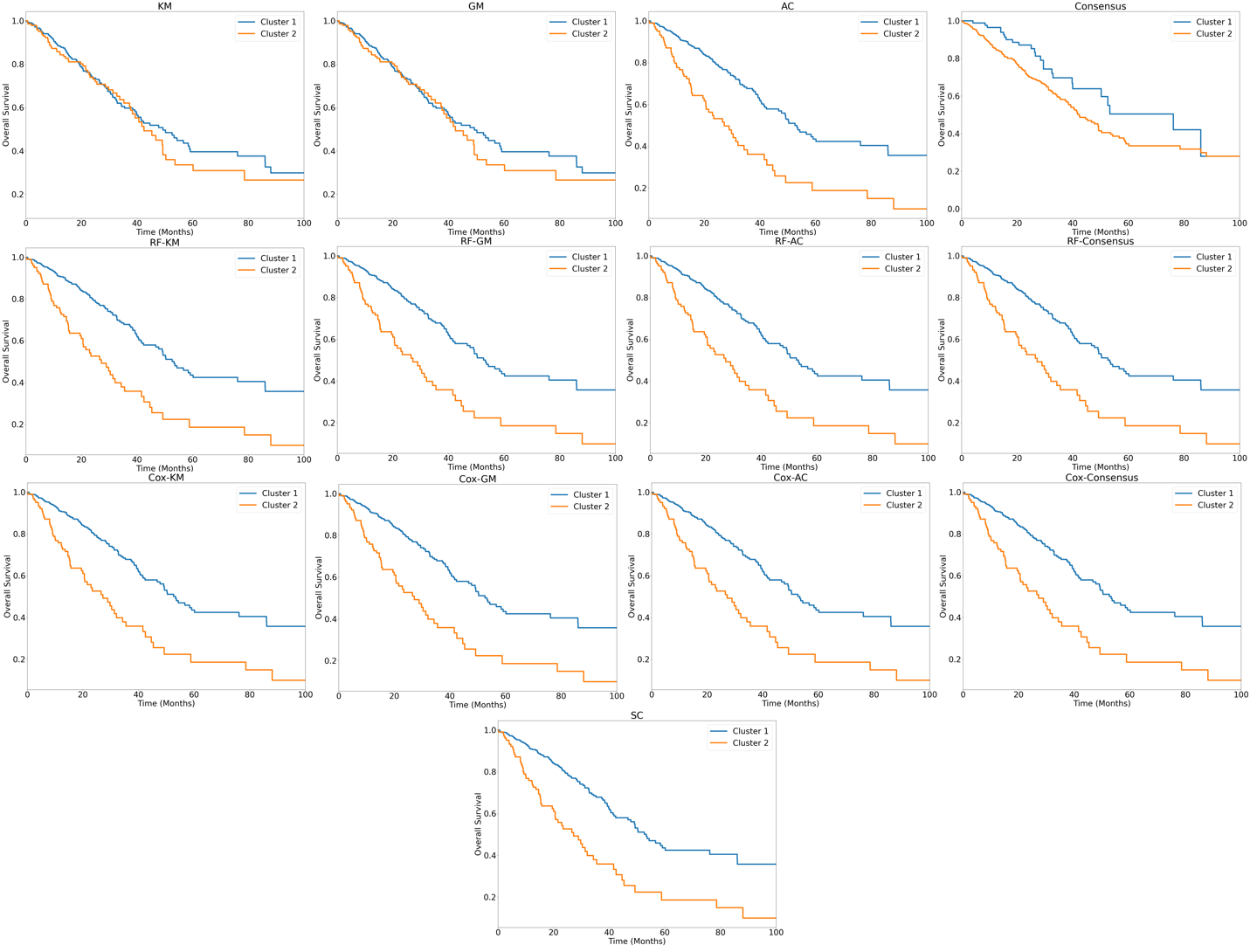


Note: These graphs display Kaplan-Meier survival curves for the two clusters identified by each of the clustering methods. One representative trial from each clustering method is presented.

**Supplementary Figure 2**

*Survival curves for different clustering methods based on Shedden data with PC features.*


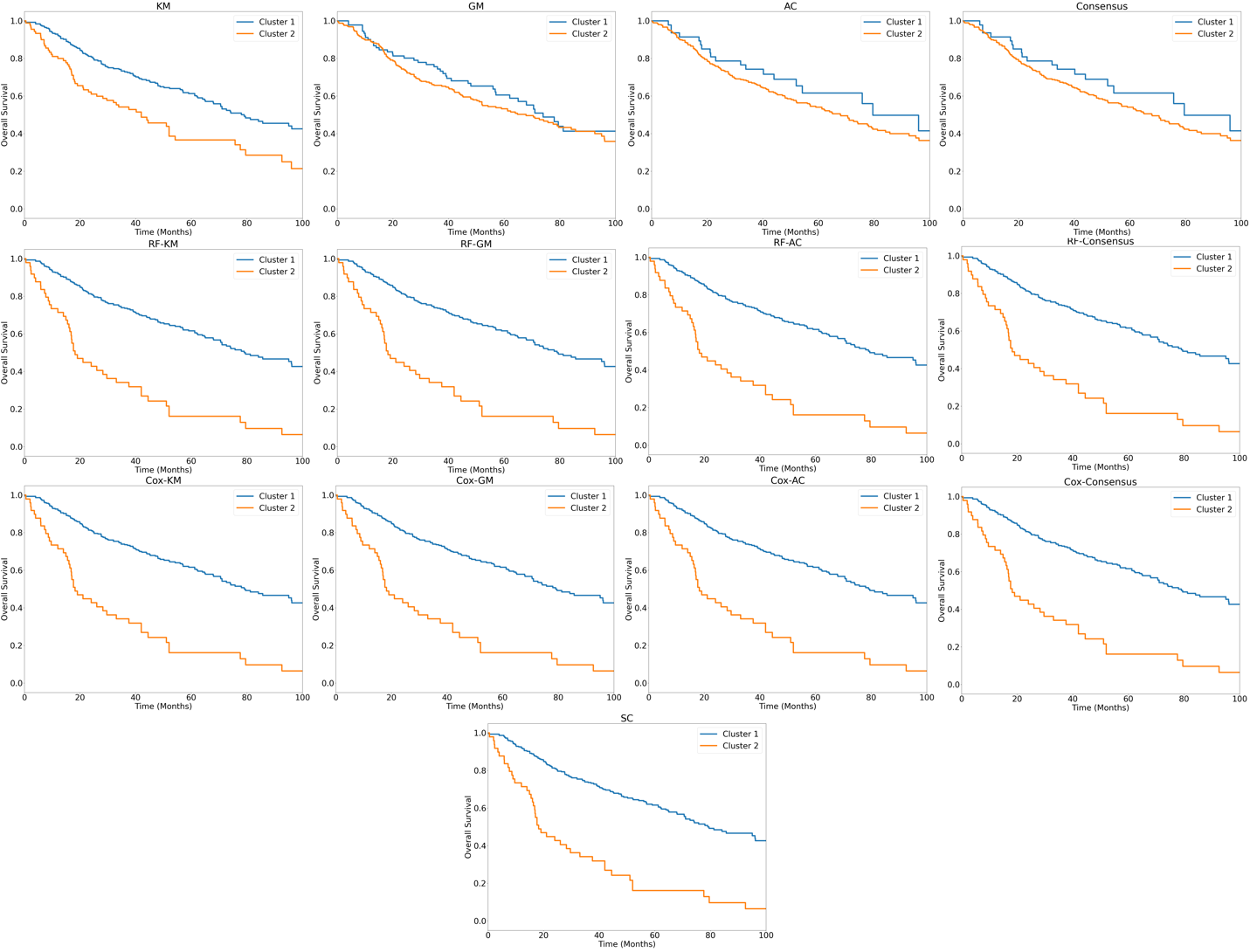


Note: These graphs display Kaplan-Meier survival curves for the two clusters identified by each of the clustering methods. One representative trial from each clustering method is presented.

**Supplementary Figure 3**

*Survival curves for different clustering methods based on TCGA-LUAD data with all the original features.*


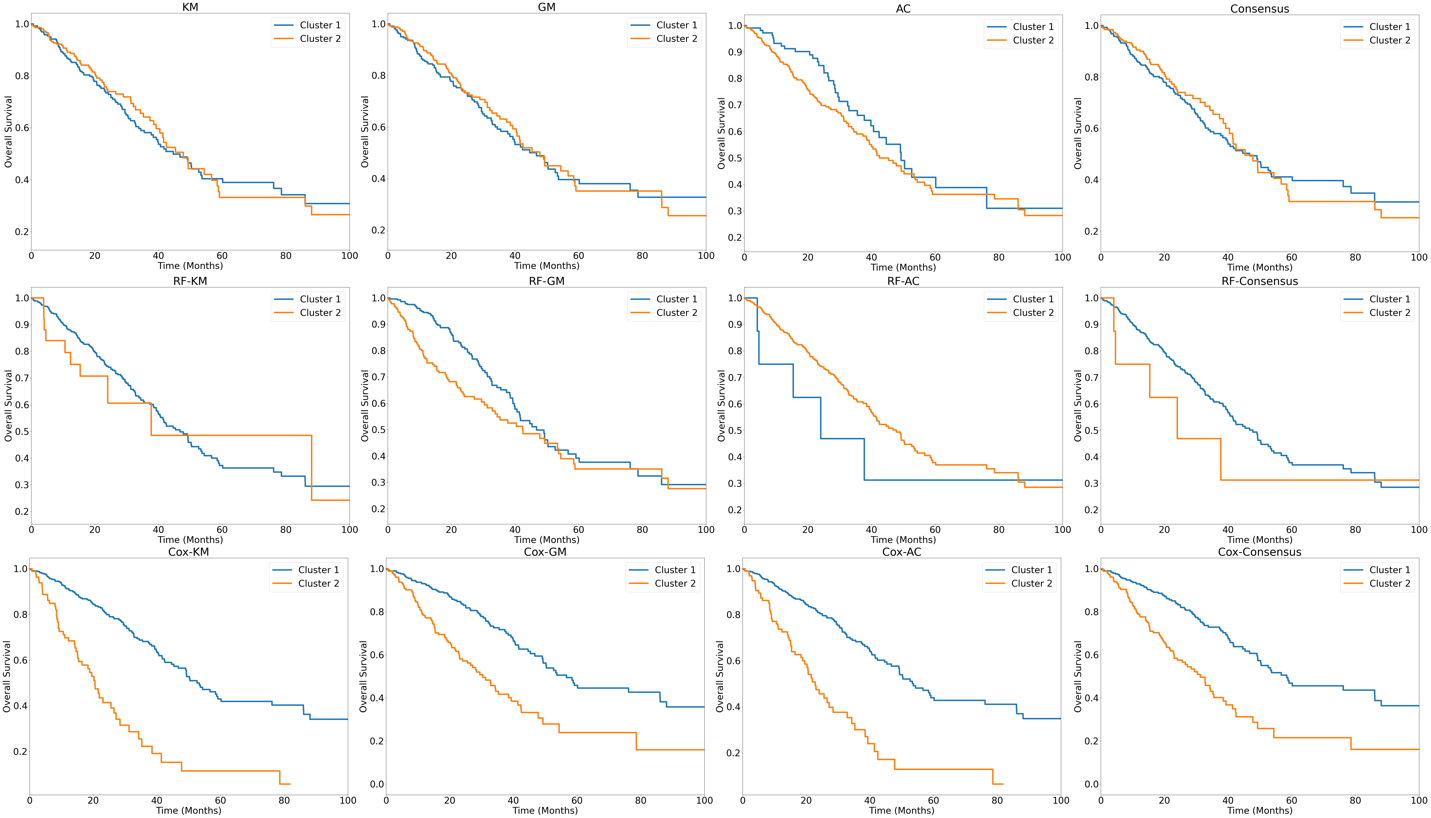


Note: These graphs display Kaplan-Meier survival curves for the two clusters identified by each of the clustering methods. One representative trial from each clustering method is presented.

**Supplementary Figure 4**

*Comparison of p-values from different clustering methods based on TCGA-LUAD data with all the original features*

Note: This forest plot displays -log_10_ p-value for comparing survival distribution between the two clusters identified by each clustering method based on TCGA-LUAD data with all the original features. The methods are grouped based on clustering algorithms, with unsupervised methods on top, semi-supervised methods in the center, and survClust (SC), the supervised method, on the bottom. Cox-AC, Cox-KM, and SC present the best performance of all the methods. The solid square indicates -log_10_(mean p-value) and horizonal line indicates the corresponding 95% CI over 200 trials for each method. The significance threshold of p=0.05 is marked with a vertical line, with significant values to the right of the line.

**Supplementary Figure 5**

*Evaluation of the consistency of clustering results from different clustering methods based on TCGA-LUAD data with all the original features*


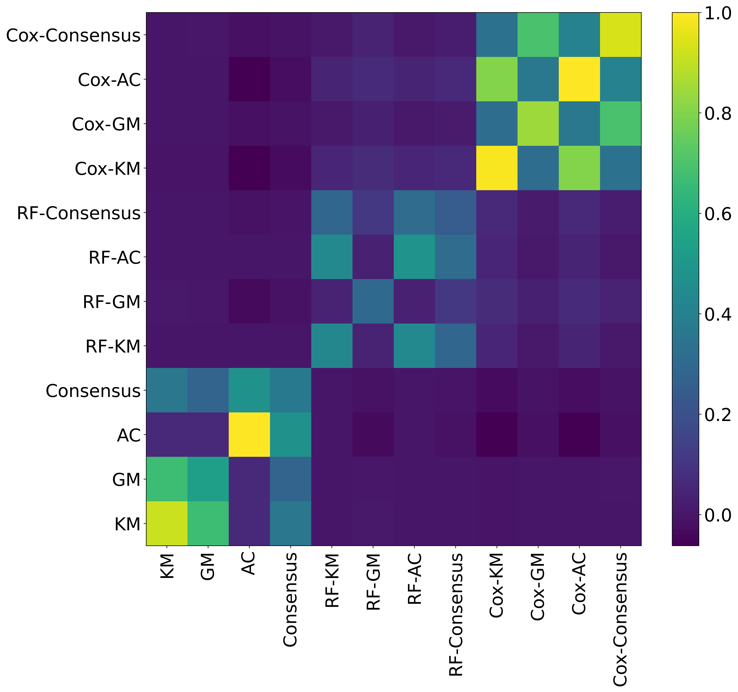


Note: The heatmap presents the ARI for each pair of clustering methods based on TCGA-LUAD data with all the original features.
